# Editorial: Alzheimer’s Dementia Recognition through Spontaneous Speech

**DOI:** 10.3389/fcomp.2021.780169

**Published:** 2021-10-21

**Authors:** Saturnino Luz, Fasih Haider, Sofia de la Fuente Garcia, Davida Fromm, Brian MacWhinney

**Affiliations:** 1 Usher Institute, Edinburgh Medical School, The University of Edinburgh, Edinburgh, United Kingdom; 2 Department of Psychology, Carnegie Mellon University, Pittsburgh, PA, United States

**Keywords:** Alzheimer’s disease, signal processing (SP), machine learning, speech processing and recognition, natural language processing (NLP), computational paralinguistics

## Editorial on the Research Topic Alzheimer’s Dementia Recognition through Spontaneous Speech

The need for inexpensive, safe, accurate and non-invasive biomarkers for Alzheimer’s disease (AD) has motivated much current research (Mandell and Green, 2011). While diagnosis and evaluation of interventions are still primarily done through clinical assessment, “digital biomarkers” have attracted increasing interest. AI-enabled speech and language analysis has emerged as promising such biomarker for the assessment of disease status (de la Fuente Garcia et al., 2020).

While a number of studies have investigated speech and language features for the detection of AD and mild cognitive impairment (Fraser et al., 2016), and proposed various signal processing and machine learning methods for this task (Petti et al., 2020), the field still lacks balanced benchmark data against which different approaches can be systematically compared. This Research Topic addresses this issue by exploring the use of speech characteristics for AD recognition using balanced data and shared tasks, such as those provided by the ADReSS Challenges (Luz et al., 2020, Luz et al., 2021). These tasks have brought together groups working on this active area of research, providing the community with benchmarks for comparison of speech and language approaches to cognitive assessment. Reflecting the multidisciplinary character of the topic, the articles in this collection span three journals: Frontiers of Aging Neuroscience, Frontiers of Computer Science and Frontiers in Psychology.

Most papers in this Reseach Topic target two main tasks: AD classification, for distinguishing individuals with AD from healthy controls, and cognitive test score regression, to infer the patient’s Mini Mental Status Examination (MMSE) score (Folstein et al., 1975). Of the twenty papers published in this collection, 14 used the ADReSS dataset (Luz et al., 2020), by itself or in combination with other data. The ADReSS dataset is a curated subset of DementiaBank’s Pitt Corpus, matched for age and gender so as to minimise risk of bias in the prediction tasks. The data consist of audio recordings of picture descriptions elicited from participants using the Cookie Theft picture from the Boston Diagnostic Aphasia Examination (Becker et al., 1994; Goodglass et al., 2001), transcribed and annotated using the CHAT coding system (MacWhinney, 2021). The papers covered a variety of approaches and models.

Antonsson et al. aimed to distinguish progressive cognitive decline from stable cognitive impairment using semantic analysis of a discourse task. Support Vector Machine (SVM) models performed best (AUC = 0.93) with both semantic verbal fluency scores and disfluency features from the discourse task. Discourse analysis revealed significantly greater use of unrelated speech in the progressive cognitive decline group compared with the stable group and healthy controls (HC).

Clarke et al. examined the impact of five different speech tasks (picture description, conversation, overlearned narrative recall, procedural recall, novel narrative retelling) on classification of 50 participants: 25 HC, 13 mild AD, 12 MCI. Linguistic features (*n* = 286) were automatically extracted from each task and used to train SVMs. Classification accuracy varied across tasks (62–78% for HC vs AD + MCI, 59–90% for HC vs AD, 50–78% for HC vs MCI) as did which features were most important to the classification.

Balagopalan et al. used linguistic and acoustic features derived from ADReSS speech and transcripts. They tuned a pretrained BERT model (Devlin et al., 2018) and compared its features to clinically-interpretable language features. The BERT model outperformed other features and achieved accuracy of 83.33% for AD classification. A ridget regressor with 25 pre-engineered features obtained root mean squared error (RMSE) of 4.56 in MMSE prediction.

Chlasta and Wolk used VGGish, a pretrained a Tensorflow model for audio feature extraction and a custom raw waveform based convolutional neural (CNN), DemCNN, to model the acoustic characteristics of AD speech on the ADreSS dataset. DemCNN provided better results than VGGish (Hershey et al., 2017) and achieved an accuracy of 62.5% using only the acoustic information.

De Looze et al. combined structural MRI, neuropsychological testing and conversational features to explore temporal characteristics of speech in a collaborative referencing task. They investigated associations with cognitive function and volumetry in brain areas known to be affected by MCI and AD. A linear mixed-effect model was built for data of 32 individuals to assess the predictive power of conversational speech features to classify clinical groups. They found that slower speech and slower turn-taking may provide useful markers for early detection of cognitive decline.

Guo et al. emphasized the importance of large normative datasets in training accurate and reliable machine learning models for dementia detection. They incorporated a new corpus of Cookie Theft picture descriptions (HC = 839, NC = 115) from the Wisconsin Longitudinal Study (Herd et al., 2014) to train a BERT model and demonstrated improved performance on the detection task compared with results of the model trained on the ADReSS data alone (82.1% vs 79.8, accuracy, and 92.3 vs 88. 3% AUC).

Haulcy and Glass investigated the use of i-vectors and x-vectors (Snyder et al., 2018), which are acoustic features originally devised for speaker identification, and linguistic features to tackle AD detection and MMSE prediction. The i-vectors and x-vectors were pre-trained on existing datasets and Random Forests, and 4.56 RMSE with a gradient boosting regressor. Linguistic and acoustic features were modelled separately. The former yielded better performance. The authors speculate that the poor performance of i-vectors and x-vectors was due to in- and out-of-domain training data mismatch.

Jonell et al. proposed a multimodal analysis of patient behavior to improve early detection of dementia. Their system captured data from clinical interviews using nine different sensor devices which recorded speech, language, facial gestures, motor signs, gaze, pupil dilation, heart rate variability and thermal emission. This information was gathered from 25 patients with AD and later combined with brain scans, psychological tests, speech therapist assessments and other clinical data. They found that multimodality, in combination with the more established biomarkers, improves clinical discrimination.

Laguarta and Subirana present an approach to the identification of different diseases which combines multiple biomarkers (features), including vocal cords, sentiment, lung and respiratory tract, among others. The authors employed transfer learning from other (non-AD) audio datasets to learn these features. The resulting model achieved up to 93% accuracy on the ADReSS dataset. Interestingly, the respiratory tract features, which were previously used in the detection of COVID-19 from a cough dataset, also proved helpful in AD detection.

Lindsay et al. investigated spontaneous speech of 78 HC and 76 AD individuals in English and French, proposing a multilingual model. Task-specific, semantic, syntactic and paralinguistic features were analysed. They found that language features, excluding task specific features, represent “generalisable” signs for cognitive language impairment in AD, outperforming all other feature sets. Semantic features were the most generalizable, with paralinguistic features showing no overlap between languages.

The work of Mahajan and Baths tested several acoustic and linguistic models, comparing their performance on ADReSS and a larger subset of DementiaBank. They employed a deep learning bimodal model to combine these features. For linguistic models, accuracy was lower on ADReSS than on DementiaBank (73 vs 88%). The authors attribute this to the smaller size of ADReSS and to overfitting in DementiaBank due to repeated samples from the same participant. Although the best linguistic model performed similarly to the bimodal learner, the authors suggest a number of possible improvements.

Martinc et al. presented a multimodal approach to AD detection using ADreSS data. The Active Data Representation method (Haider et al., 2020) was used for fusion of acoustic and textual features at sentence and word level, along with temporal aspects of linguistic features. They achieved an accuracy of 93.75% through late fusion of acoustic, text and temporal models.

Meghanani et al. compared two approaches to the challenge tasks based on use of the non-automatic, hand-created transcripts. Both methods relied on the extraction of n-grams of varying lengths (*n* = 2,3,4, and 5) from the transcripts. The first method employed CNNs with a single convolutional layer in which the kernel size was adapted to the n-gram size. The second method used the fastText model with bigrams and trigrams. The fastText models outperformed the CNN models, achieving 83.3% accuracy for classification and RMSE of 4.87 for prediction of MMSE scores.

Millington and Luz approached the data representation problem in the ADReSS dataset by converting its text transcriptions into word co-occurrence graphs and computing several graph structure metrics. They found that AD graphs have lower heterogeneity and centralization, but higher edge density. These metrics were used as input features to standard machine learning classifiers and regressors. A graph embedding metric was tested for comparison. Graph metrics outperformed graph embedding, achieving 66.7% accuracy in classification, and a 5.67 RMSE in MMSE regression.

Nasreen et al. investigated the role of conversational features such as dysfluencies, pauses, overlaps and other interactional elements in AD detection. They used the Carolinas Conversations Collection (Pope and Davis, 2011) to create classification models based on those features. The combination of dysfluency and interactional features resulted in a classification accuracy of 90%. These findings in conversational speech seem to agree with the findings from other papers in this Research Topic, which highlighted the importance of pauses and dysfluency in detecting AD in the ADReSS monologue data.

Parvin et al. performed a randomised controlled clinical trial to investigate the effects of dual-task training on 26 patients with AD. Patients performed physical, cognitive and mental assessments and had their brain oscillations measured pre- and post-intervention, which consisted of a 12-weeks visual training program. The trained group showed significant improvements in cognitive function, mood and fitness. This was associated with a significant positive change in brain oscillation.

Sadeghian et al. examined the potential of an almost fully automated system for AD detection. Rather than using DementiaBank, they collected 72 new samples (26 AD, 46 HC) with higher quality audio. ASR was performed on data with pauses removed using voice activity detection. From this, they extracted 236 textual features and then used a genetic algorithm as well as a Multi-Layer Perceptron to identify the 10 most useful features, achieving 94% accuracy in detection.

Shah et al. used speech samples from the DementiaBank database for binary classification and MMSE regression. Although they developed models that combined acoustic and language-based features, their best performing model for binary classification used language-based features only with a regularized logistic regression, achieving 85.4% accuracy on a hold-out test set. A more reduced set of language features was their best performing model for the regression task, with an RMSE of 5.62.

Yuan et al. presented a method for encoding filled and unfilled pauses in transcripts to fine tune the training of language models using BERT and ERNIE. The accuracy of dementia detection improved to 89.6% (with ERNIE). Compared with controls, the individuals with dementia vocalised filled pause *um* much less frequently than *uh,* and their language samples included more pauses.

Zhu et al. used a transfer learning technique to fine-tuning the last layers of a pretrained model with customized layers for AD detection. The MobileNet and YAMNet network architectures were employed for this. They then used speech and text versions of BERT, individually and in combination for the same task. The text models outperformed the speech models, with the version based on pre-training with the longest input frame achieving 89. 58% accuracy. The models which combined audio and speech data generally performed better than the models separately.

The studies in this Research Topic represent the state of the art in dementia detection, and contribute to the increasing body of evidence supporting machine learning and spoken language for detecting cognitive decline.
